# Longitudinal Changes of Handgrip, Knee Extensor Muscle Strength, and the Disability of the Arm, Shoulder and Hand Score in Cardiac Patients During Phase II Cardiac Rehabilitation

**DOI:** 10.3390/diseases7010032

**Published:** 2019-03-26

**Authors:** Kazuhiro P. Izawa, Yusuke Kasahara, Koji Hiraki, Yasuyuki Hirano, Koichiro Oka, Satoshi Watanabe

**Affiliations:** 1 Department of Public Health, Graduate School of Health Sciences, Kobe University, Kobe 654-0142, Japan; izawapk@harbor.kobe-u.ac.jp; 2 Cardiovascular stroke Renal Project (CRP), Kobe 654-0142, Japan; kasahara.y@marianna-u.ac.jp (Y.K.); hiraki7@marianna-u.ac.jp (K.H.); hirano@tks.bunri-u.ac.jp (Y.H.); koka@waseda.jp (K.O.); 3 Department of Rehabilitation Medicine, St. Marianna University School of Medicine Yokohama-city Seibu Hospital, Yokohama 241-0811, Japan; 4 Department of Rehabilitation Medicine, St. Marianna University School of Medicine Hospital, Kawasaki 216-8511, Japan; 5 Department of Physical Therapy, Tokushima Bunri University, Tokushima 770-8514, Japan; 6 Faculty of Sport Sciences, Waseda University, Saitama 359-1192, Japan

**Keywords:** cardiac surgery, DASH, handgrip, knee extensor muscle strength, phase II cardiac rehabilitation

## Abstract

Handgrip strength (HS) and knee extensor muscle strength (KEMS) showed a negative correlation with the Disabilities of the Arm, Shoulder, and Hand (DASH) score at one month following cardiac surgery. We performed a longitudinal study to examine changes in HS/KEMS and DASH score during phase II cardiac rehabilitation (CR) in patients after cardiac surgery. We measured and assessed HS, KEMS, and DASH score in 41 consecutive patients at one and three months following cardiac surgery and examined the relation between these factors at three months following cardiac surgery. Wilcoxon signed-rank test and Spearman correlation coefficients were used to analyze the results. Finally, 26 patients (63.2 years, 73.1% male) were analyzed. There were significant differences from one month to three months following cardiac surgery in HS (26.78 ± 8.26 to 31.35 ± 9.41 kgf, *p* < 0.001), KEMS (1.53 ± 0.42 to 1.72 ± 0.46 Nm/kg, *p* = 0.001), and DASH score (14.76 ± 12.58 to 7.62 ± 9.29, *p* < 0.001). DASH score correlated negatively with HS (r = −0.41, *p* = 0.01) but not with KEMS (r = −0.32, *p* = 0.09) after three months of phase II CR. Although HS, KEMS, and DASH scores changed significantly from one to three months following cardiac surgery during phase II CR, only HS correlated negatively with DASH score at three months following cardiac surgery.

## 1. Introduction

The Disabilities of the Arm, Shoulder, and Hand (DASH) questionnaire is a reliable and previously validated patient-reported outcome measure which can be used to assess self-reported upper extremity symptoms and disabilities in relation to having surgical treatment for a variety of upper extremity conditions [1,2,3,4]. A previous report suggested that postoperative muscle weakness was associated with inflammation immediately after cardiac surgery [5]. In addition, post-cardiac surgery patients need to follow sternal precautions to protect their sternum after open-heart surgery [6]. Muscle weakness may result from both the limitation of upper extremity movement and infection in these patients. Therefore, upper extremity muscle strength may limit patients’ activities of daily living. Moreover, the items in the DASH questionnaire to no small extent include not only upper extremity function but also its relation to lower extremity function [1,2].

We previously reported on our cross-sectional investigation on the relationship between handgrip and knee extensor muscle strength and the DASH score of cardiac surgery patients at one month following surgery (74.4% men, age: 62.1 years) [7] and found that both strength factors correlated negatively with the DASH score [7]. In that study, we proposed that the DASH score could be valuable in assessing post-cardiac surgery patients’ handgrip and knee extensor muscle strength as a representative measure of upper and lower extremity function [7].

On the other hand, previous studies suggested that upper and lower extremity muscle strength predicted cardiac death, all-cause death in patient cardiac disorders such as heart failure [8,9]. The various goals of cardiac rehabilitation (CR) in patients after cardiac surgery include improvement of muscle strength, exercise capacity, and health-related quality of life, and reduction of coronary risk factors, cardiac events, sudden death, all-cause mortality, and costs of hospitalization [10,11,12,13]. The findings reported on handgrip and knee extensor muscle strength in cardiac surgery patients supported the improvements that can be attained with phase II CR [11,14]. Thus, we believe that DASH scores would also change longitudinally after cardiac patients participate in phase II CR.

We hypothesized that not only handgrip and knee extensor muscle strength but also DASH score would change from one month to three months following cardiac surgery in patients participating in phase II CR. However, it is unknown whether the longitudinal change in DASH scores, and handgrip and knee extensor muscle strength in these patients would directly correlate with the DASH score at three months following cardiac surgery.

The present study aimed to evaluate: (1) differences in the longitudinal change in handgrip and knee extensor muscle strength, and DASH score from one month to three months following cardiac surgery, and (2) to ascertain the relationship between handgrip and knee extensor muscle strength, and DASH scores at three months following cardiac surgery in cardiac patients participating in phase II CR.

## 2. Materials and Methods

### 2.1. Participants

This was a longitudinal study of 41 consecutive patients who underwent surgeries including valve replacement or coronary artery bypass grafting and visited a university hospital outpatient clinic 1 month after surgery and were referred to the Department of Rehabilitation Medicine for initial evaluation of handgrip and knee extensor muscle strength, and DASH score.

Patients were excluded if they had peripheral vascular, neurological, orthopedic, or pulmonary disease or advanced renal disease or were on dialysis. Medical records were reviewed to evaluate the following patient characteristics: Age, sex, body mass index (BMI), left ventricular ejection fraction (LVEF), and disease etiology. Handgrip and knee extensor muscle strength, and DASH score were first evaluated at 1 month following cardiac surgery, and then during phase II CR, at 3 months following cardiac surgery. The present study complied with the principles of the Declaration of Helsinki regarding investigations in humans and was approved by the local institutional review board of our university.

### 2.2. Handgrip

We evaluated handgrip strength with a standard JAMAR dynamometer with an adjustable handle (Bissell Healthcare Co., Grand Rapids, MI, USA) as the index of upper limb muscle power. All patients used the dynamometer at the second grip position [10,11,14,15]. To measure handgrip strength, subjects were seated with their shoulder adducted and neutrally rotated, elbow flexed at 90°, forearm in the neutral position, and wrist between 0° and 30° of dorsiflexion and between 0° and 15° of ulnar deviation. We made three measurements each on both hands while taking care to avoid a possible Valsalva effect. After the measurements were completed, the index of handgrip strength was calculated as the average of the highest value of the right-hand plus left-hand grip strength and dividing by two.

### 2.3. Knee Extensor Muscle Strength

We used a Biodex System 2 isokinetic dynamometer (Biodex Medical Systems, Inc., New York, NY, USA) to measure knee extensor muscle strength as the index of lower limb muscle power. Patients performed up to five repetitions of knee extension at isokinetic speeds of 60°/s so that the knee extension muscular strength peak torque per body weight value of both knees could be measured. Biodex System 2 software analyzed the isokinetic test results [10,11,14,15]. Following these measurements, we obtained the index of knee extension muscular strength by averaging the highest value of the right-side plus left-side knee extension muscular strength and dividing by 2 [10,11,14,15].

### 2.4. DASH

We used the Japanese version of the DASH questionnaire, whose validity, reliability, and responsiveness were previously evaluated in Japanese patients, to examine the health status of each patient for the preceding week via the questionnaire’s 30-item disability/symptom scale [1,2,3,4]. The items measure the difficulty patients experience when performing various physical activities with the problem being that of the hand, shoulder, or arm and evaluate the severity of each of the symptoms of pain, activity-related pain, tingling, weakness, and stiffness as well as the effect of the problems on the patient’s work, social activities, sleep, and self-image. There are five responses for each item. The scores for all responses are then totaled to compute the DASH score, which ranges from 0 (no disability) to 100 (most severe disability) [1,2,3,4].

### 2.5. Phase II CR

The supervised-recovery phase II CR outpatient program was completed from 1 to 3 months (8 weeks) following cardiac surgery in the present study. In it, patients underwent 1 h of supervised aerobic and resistance exercise performed once or twice a week. Each session included a warm-up period (10 min), resistance training (20 min), aerobic exercise (20 min), and a cooldown period (10 min). In the first session, patients performed a series of upper and lower limb, and body stretches before and after the exercise [10,11,14,15]. During the resistance training, three to five sets of a series of two upper limb exercises were performed with iron weights and/or handgrip devices at a resistance allowing the patient to complete five repetitions with a perceived exertion rating of 11–13 on the 6–20 Borg scale. Aerobic exercise at exercise intensity was performed by walking on a treadmill or riding a cycle ergometer to maintain the heart rate at the anaerobic threshold using a rating of perceived exertion of 11–13 on the Borg scale [10,11,14,15].

### 2.6. Statistical Analysis

Results relating to clinical characteristics are expressed as mean ± standard deviation (SD) and percent. We analyzed longitudinal changes of handgrip and knee extensor muscle strength, and DASH score from 1 month to 3 months following cardiac surgery with the non-parametric Wilcoxon signed-rank test because the sample size was very small. Relations between DASH scores and both handgrip strength and knee extensor muscle strength at 3 months following cardiac surgery were tested via Spearman’s rank correlation coefficient analysis. A *p* value of <0.05 was considered to be statistically significant. All statistical analyses were conducted with IBM SPSS 24.0 J statistical software (IBM SPSS Japan, Inc., Tokyo, Japan).

## 3. Results

### 3.1. Patients’ Clinical Characteristics

Patient characteristics are presented in Table 1. Of the 41 patients, 15 were excluded because of patient convenience, inability to come to the hospital to evaluate these values, patient’s request, no measurement of handgrip and/or knee extensor muscle strength values, or an incompletely evaluated DASH questionnaire at three months following cardiac surgery. Thus, the final analysis comprised 26 patients (male, 73.1%; age, 63.2 ± 7.9 years).

### 3.2. Longitudinal Changes in Handgrip and Knee Extensor Strengths, and DASH Score

During the phase II CR, there were significant differences from one month to three months following cardiac surgery in handgrip strength (from 24.6 ± 8.3 to 31.4 ± 9.4 kgf, *p* < 0.001), knee extensor muscle strength (from 1.4 ± 0.4 to 1.7 ± 0.5 Nm/kg, *p* < 0.001), and DASH score (from 14.8 ± 12.5 to 7.6 ± 9.3, *p* < 0.001) (Table 2).

### 3.3. Handgrip and Knee Extensor Strength, and DASH score at 3 Months Following Cardiac Surgery

**Figure 1:**
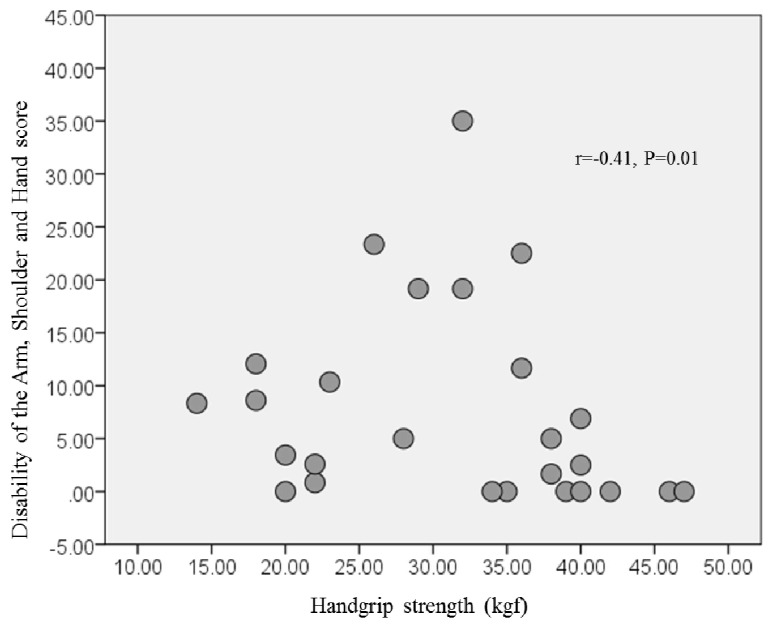
Relationship between handgrip strength and DASH scores of the patients at 3 months following cardiac surgery. DASH, Disabilities of the Arm, Shoulder, and Hand.

Figure 1 shows the relation between handgrip strength and the DASH score at three months following cardiac surgery. The DASH score correlated negatively with handgrip strength (r = −0.41, *p* = 0.01) but did not correlate with knee extensor muscle strength (r = −0.32, *p* = 0.09).

## 4. Discussion

This is the first time, to our knowledge, that the relationship between the DASH score and both handgrip and knee extensor muscle strength has been evaluated in post-cardiac surgery patients during phase II CR. This study found that the DASH scores were lower at three months than at one month following cardiac surgery during phase II CR. However, in comparing the decrease in DASH score from one month to three months, the DASH score at three months following cardiac surgery correlated negatively only with handgrip strength. In other words, the DASH score improved along with both handgrip and knee extensor strength, however, it was that the relationship between DASH and knee extensor is insignificant (*p* = 0.09) in the present study.

Among the benefits of phase II CR outpatient programs following cardiac surgery, physiological outcomes such as peak oxygen uptake, handgrip, knee extensor muscle strength, and maximum phonation time were the focus of many previous reports [10,11,14,15,16]. In the present study, handgrip and knee extensor muscle strength improved significantly from one to three months during phase II CR (Table 2). We also previously reported improvements in both handgrip and knee extensor muscle strength similar to those found in the present study in cardiac patients undergoing phase II CR outpatient programs [11,15]. With regard to physiological outcomes, these previous findings may support our current results.

There was a negative correlation between handgrip strength and DASH score at three months following cardiac surgery in the present study, however there was no correlation with knee extensor muscle strength. Several reasons could explain these findings in our post-cardiac surgery patients.

A previous study [17] suggested that in comparison with the fittest subjects and cardiac patients, male subjects achieving a peak exercise capacity of <5 METs (metabolic equivalent of task) have age-adjusted relative risks of death for each of the major risk factors. In another study, an exercise capacity of ≥5 METs in male heart failure patients was found to be equivalent in cardiac patients with approximate strength values of 35.2 kgf for handgrip strength and 1.70 Nm/kg for knee extensor muscle strength [18]. Handgrip strength (31.35 kgf) at three months following cardiac surgery in the present study was below the above target variable related to an exercise capacity of five METs in chronic heart failure patients and thus, as an indicator of upper limb function, this value is still not adequate. Therefore, there is room for improvement in handgrip strength after three months in cardiac surgery patients.

In contrast, knee extensor muscle strength (1.72 Nm/kg) was slightly higher than the above target variable related to an exercise capacity of 5 METs. Despite the difference in etiology between our study patients and that of the patients with chronic heart failure, the present study indicated that handgrip and knee extensor muscle strength after cardiac surgery may be affected in patients at risk for poor muscle strength, thus supporting the findings of these previous studies. Handgrip and knee extensor muscle strengths differ between males and females [19]. Because 73.1% of the patients in the present study were male, we need to evaluate differences in sex in a more equal manner in future trials.

The DASH score was designed to measure physical function and symptoms related to musculoskeletal disorders of the arms [1,2,3,4]. The items of DASH score are based on the degree of difficulty in performing different physical activities with the problem arm, shoulder, or hand; the severity of each of the symptoms of pain, activity-related pain, tingling, weakness, and stiffness; and the effect of the problem on social activities, work, sleep, and self-image [3,4].

The deterioration of skeletal muscle due to surgery, which occurs rapidly during the postoperative period, is a morbidity with serious complications [5]. In addition, after cardiac surgery, medical staff may require their patients to follow sternal precautions, such as a method to protect their sternum after open-heart surgery [6]. All patients after this surgery may follow these precautions to avoid dehiscence of the sternum as it is healing [6], to protect the patient and prevent possible infections.

Therefore, handgrip weakness may be a result of limitations in upper extremity activity. This might be one reason for the negative correlation between DASH scores and upper extremity strength in this study. Our results indicate that in patients at three months following cardiac surgery who have completed phase II CR, the DASH score may be valuable in assessing those patients with a potentially poor physiological outcome, especially when handgrip strength is used as the index of upper extremity function as it may be easier to check over time than lower extremity function. It follows that the present results would also support an emphasis during phase II CR on upper extremity strength as it relates to activities of daily living in these patients.

There are several limitations in this study. The sample size was very small, we did not determine sample size, and only a few female cardiac surgery patients were included. Analysis of sex-related differences in DASH scores in female cardiac surgery patients is required. We could not evaluate age-related differences in DASH scores in the cardiac surgery patients during phase II CR. Baseline handgrip and knee extensor muscle strengths were reported to be lower in elderly patients 65 years and older than in middle-aged patients under 65 years old who participated in an exercise-based, supervised, recovery-phase II CR outpatient program [15]. This previous study also found that age-related differences in various physiologic measures resulted in greater improvement in the middle-aged vs. older-aged patients [15]. We did not evaluate the effects of postoperative complications, the time patients entered the earlier phase I CR program, or a longer hospital stay on selection bias related to DASH score testing. We also did not evaluate the effect of occupational therapy or other rehabilitation programs, which were not the same for each patient. 

Finally, due to the limited availability of related data, we did not assess timing of the DASH evaluations in relation to clinical characteristics, cardiac-related mortality, and re-hospitalization. Those are several important links that are missing in the present study. Both the functional and prognostic values of handgrip, and where the DASH score may align are missing. This is the case for knee extensor muscle strength as well. These deficiencies should be corrected in future longitudinal studies.

## 5. Conclusions

Longitudinal differences were found between handgrip and knee extensor muscle strength, and DASH scores at one and three months following cardiac surgery in patients participating in a phase II CR program. Following phase II CR, not only handgrip and knee extensor muscle strength but also DASH scores improved in all of the cardiac surgery patients. However, handgrip strength correlated negatively with DASH scores at three months following cardiac surgery. This relatively short-term study will require long-term follow-up data to evaluate whether the DASH score can be useful in assessing patients with a potentially poor physiological outcome who would benefit from a targeted CR outpatient program. Based on our results, this might be a more useful and simpler tool for those entering rehabilitation to help identify those with lower muscle strength.

## Figures and Tables

**Table 1:** Patients’ clinical characteristics.

No. of patients	26
Age (y)	63.2 ± 7.9
Sex (%, male)	73.1
BMI (kg/m^2^)	22.6 ± 3.1
LVEF (%)	52.7 ± 12.8
Etiology (%)	
CABG	61.5
Valve replacement	38.5
Medications (%)	
Βeta-blockers	50.0
ACEI/ARB	50.0
Diuretic	73.1

ARB, angiotensin receptor blocker; ACEI, angiotensin converting enzyme inhibitor; BMI, body mass index; CABG, coronary artery bypass grafting; LVEF, left ventricular ejection fraction.

**Table 2:** Longitudinal changes of handgrip strength, knee extensor muscle strength, and DASH scores of the cardiac patients.

Values	1 Month	3 Months	Test Statistic	*p* Value
Handgrip strength (kgf)	26.78 ± 8.26	31.35 ± 9.41	4.25	<0.001
Knee extensor muscle strength (Nm/kg)	1.53 ± 0.42	1.72 ± 0.46	3.21	0.001
DASH score	14.76 ± 12.58	7.62 ± 9.29	–3.24	0.001

DASH, Disabilities of the Arm, Shoulder, and Hand.

## References

[1] Imaeda T., Uchiyama S., Wada T., Okinaga S., Sawaizumi T., Omokawa S., Momose T., Moritomo H., Gotani H., Abe Y. (2010). Clinical Outcomes Committee of the Japanese Orthopaedic Association and the Functional Evaluation Committee of the Japanese Society for Surgery of the Hand. Reliability, validity, and responsiveness of the Japanese version of the Patient-Rated Wrist Evaluation. J. Orthop. Sci..

[2] Imaeda T., Toh S., Nakao Y., Nishida J., Hirata H., Ijichi M., Kohri C., Nagano A. (2005). Impairment Evaluation Committee, Japanese Society for Surgery of the Hand. Validation of the Japanese Society for Surgery of the Hand version of the Disability of the Arm, Shoulder, and Hand questionnaire. J. Orthop. Sci..

[3] Beumer A., Lindau T.R. (2014). Grip strength ratio: A grip strength measurement that correlates well with DASH score in different hand/wrist conditions. BMC Musculoskelet. Disord..

[4] Gummesson C., Atroshi I., Ekdahl C. (2003). The disabilities of the arm, shoulder and hand (DASH) outcome questionnaire: Longitudinal construct validity and measuring self-rated health change after surgery. BMC Musculoskelet. Disord..

[5] Iida Y., Yamazaki T., Kawabe T., Usui A., Yamada S. (2014). Postoperative muscle proteolysis affects systemic muscle weakness in patients undergoing cardiac surgery. Int. J. Cardiol..

[6] Sears B. Sternal Precautions after Open Heart Surgery. https://www.verywell.com/sternal-precautions-2696084.

[7] Izawa K.P., Kasahara Y., Hiraki K., Hirano Y., Watanabe S. (2017). Relation between the Disability of the Arm, Shoulder and Hand Score and muscle strength in post-cardiac surgery patients. Diseases.

[8] Hülsmann M., Quittan M., Berger R., Crevenna R., Springer C., Nuhr M., Mörtl D., Moser P., Pacher R. (2004). Muscle strength as a predictor of long-term survival in severe congestive heart failure. Eur. J. Heart Fail..

[9] Pavasini R., Serenelli M., Celis-Morales C.A., Gray S.R., Izawa K.P., Watanabe S., Colin-Ramirez E., Castillo-Martínez L., Izumiya Y., Hanatani S., Onoue Y. (2018). Grip strength predicts cardiac adverse events in patients with cardiac disorders: An individual patient pooled meta-analysis. Heart.

[10] Izawa K., Hirano Y., Yamada S., Oka K., Omiya K., Iijima S. (2004). Improvement in physiological outcomes and health-related quality of life following cardiac rehabilitation in patients with acute myocardial infarction. Circ. J..

[11] Hirano Y., Izawa K., Watanabe S., Yamada S., Oka K., Kasahara Y., Omiya K. (2005). Physiological and health-related quality of life outcomes following cardiac rehabilitation after cardiac surgery. J. Jpn. Phys. Ther. Assoc..

[12] Hirschhorn A.D., Richards D.A., Mungovan S.F., Morris N.R., Adams L. (2012). Does the mode of exercise influence recovery of functional capacity in the early postoperative period after coronary artery bypass graft surgery? A randomized controlled trial. Interact. Cardiovasc. Thorac. Surg..

[13] Anderson L., Thompson D.R., Oldridge N., Zwisler A.D., Rees K., Martin N., Taylor R.S. (2016). Exercise-based cardiac rehabilitation for coronary heart disease. Cochrane Database Syst. Rev..

[14] Izawa K.P., Watanabe S., Oka K., Hiraki K., Morio Y., Kasahara Y., Osada N., Omiya K., Makuuchi H. (2011). Cardiac rehabilitation outcome following percutaneous coronary intervention compared to cardiac surgery. Recent Pat. Cardiovasc. Drug Discov..

[15] Izawa K.P., Watanabe S., Oka K., Hiraki K., Morio Y., Kasahara Y., Osada N., Omiya K., Iijima S. (2010). Age-related differences in physiologic and psychosocial outcomes after cardiac rehabilitation. Am. J. Phys. Med. Rehabil..

[16] Izawa K.P., Kasahara Y., Hiraki K., Hirano Y., Watanabe S. (2017). Age-related differences of maximum phonation time in patients after cardiac surgery. Diseases.

[17] Myers J., Prakash M., Froelicher V., Do D., Partington S., Atwood J.E. (2002). Exercise capacity and mortality among men referred for exercise testing. N. Engl. J. Med..

[18] Izawa K.P., Watanabe S., Oka K., Hiraki K., Morio Y., Kasahara Y., Watanabe Y., Katata H., Osada N., Omiya K. (2012). Upper and lower extremity muscle strength levels associated with an exercise capacity of 5 metabolic equivalents in male patients with heart failure. J. Cardiopulm. Rehabil. Prev..

[19] Izawa K.P., Oka K., Watanabe S., Yokoyama H., Hiraki K., Morio Y., Kasahara Y., Omiya K. (2008). Gender-related differences in clinical characteristics and physiological and psychosocial outcomes of Japanese patients at entry into phase II cardiac rehabilitation. J. Rehabil. Med..

